# Mechanical regulation of mesenchymal stem cell osteogenesis, bone matrix homoeostasis, and skeletal pathology

**DOI:** 10.1177/20417314261454194

**Published:** 2026-06-04

**Authors:** Udipt R. Das, Hussain Jaffery, Penelope M. Tsimbouri, Matthew J. Dalby

**Affiliations:** 1 Centre for the Cellular Microenvironment, School of Molecular Biosciences, Advanced Research Centre, College of Medical, Veterinary and Life Sciences, University of Glasgow, UK

**Keywords:** MSC, mechanotransduction, osteoblasts, osteoclasts, remodelling, inflammation, nanovibration

## Abstract

Osteogenesis is the process by which mesenchymal stem/stromal cells (MSCs) differentiate into mature osteoblasts, forming a mineralised bone matrix. This process is regulated by soluble factors, mechanical stimuli, and the extracellular matrix (ECM), which together maintain bone and mineral homoeostasis. Mechanotransduction, the conversion of physical cues into intracellular signals, is crucial for MSC fate determination and orchestrates bone matrix remodelling, balancing formation and resorption. Continuous mechanical loading supports optimal osteogenic differentiation, whereas mechanical unloading (sub-physiological mechanical loading) disrupts this equilibrium and increases bone loss risk. These processes involve complex crosstalk among local and systemic factors, immune cells, and osteoblast-osteoclast interactions in response to mechanical cues. This review discusses key biomechanical factors regulating MSC osteogenic differentiation and bone remodelling, and synthesises evidence on skeletal immobilisation and other unloading-associated conditions that contribute to skeletal anomalies. It further emphasises nanovibrational stimulation as a novel approach to enhance MSC osteogenesis and mitigate skeletal anomalies.

## Graphical abstract

**Graphical Abstract:**
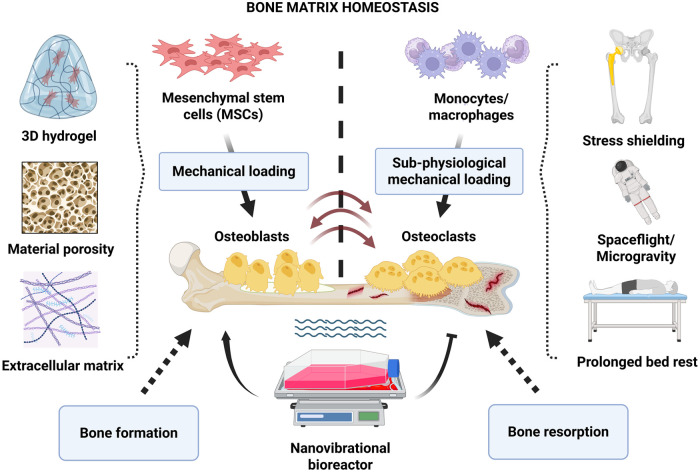

## Introduction

Bone is a rigid connective tissue that offers protection to internal organs, supports muscles for locomotion, and, importantly, contains red bone marrow, which harbours multipotent stem cells, such as mesenchymal stem/stromal cells (MSCs) and haematopoietic stem cells (HSCs), as well as their lineage-associated progeny. It also aids in maintaining mineral balance via extensive remodelling, making it a dynamic metabolic reservoir.^1^ Focussing on mesenchymal stem cells, they represent a heterogeneous population of multipotent progenitors, also referred to as mesenchymal stromal cells.^1,2^ They possess the capacity to differentiate into osteoblastic, chondrogenic, reticular and adipogenic cells and have been widely studied for their immunomodulatory and regenerative properties, including their ability to relocate to sites of tissue damage and foster regeneration.^2^ The ability to maintain and tune MSC multipotency is important for eventual therapeutic use.^3^

MSC fate is regulated by both biophysical and biochemical cues, including extracellular matrix (ECM), hypoxia, and certain soluble factors.^4–6^ ECM proteins, such as laminin (LAM), collagen (COL), and fibronectin (FN), are secreted by MSCs or other stromal cells, facilitating MSC adhesion and participating in lineage specification via integrin-directed signalling.^5^ Additionally, soluble factors, including cytokines, growth factors, and chemokines, are known to induce MSC proliferation, motility, and morphogenesis.^4^ Autologous or allogenic cell transplantation represents a promising strategy to enhance bone regeneration. This approach exploits MSC-derived osteoblasts to treat fractures, osteoporosis, and a plethora of skeletal pathologies.^7^

In addition to biochemical cues, MSCs are sensitive to the mechanical properties of their microenvironment. Substrate stiffness or elasticity, which is the ability of materials to resist deformation and return to their original state, and viscoelasticity, or the time-dependent deformation synchronising viscous and elastic behaviour, represent key determinants of MSC lineage commitment both in vitro and in vivo.^8–10^ Further, exposure to exogenous mechanical cues within defined physiological conditions can bias MSC differentiation towards osteogenesis or adipogenesis.^9^ These substrate-derived or exogenous mechanical cues are transduced through integrin-focal adhesion complexes, intracellular cytoskeletal tension, and multiple mechanosensitive (or mechanotransductive) pathways such as RhoA-Rho-associated coiled-coil containing protein kinase (RhoA/ROCK) and Yes-associated protein/transcriptional coactivator with PDZ binding motif (YAP/TAZ) signalling, underscoring the significance of mechanotransduction in bone regeneration and tissue engineering applications.^10^

In this context, this review adopts a narrative approach to synthesise current research on the role of mechanical stimulation in MSC osteogenesis and bone matrix remodelling. The objective is to integrate, critically examine, and acknowledge key mechanobiological mechanisms across diverse experimental models and loading conditions, rather than to provide a systematic evaluation of all available studies. Relevant literature was identified through searches of databases including PubMed, Google Scholar, and Scopus using keywords related to mechanotransduction, MSC osteogenic differentiation, inflammation, and bone remodelling.

## Mechanostimulation and osteogenesis

Osteogenesis, which refers to the overall process of bone formation, and osteoblastogenesis, which specifically describes the differentiation of mesenchymal progenitors into osteoblasts, are intricate mechanisms in which MSCs serve as osteoblast precursors, coordinated by mechanical cues, signalling cascades, pathway crosstalk, and interactions with the extracellular matrix.^11,12^ Cellular fate in general, and osteogenic fate in particular, is a measure determined by how cells perceive and respond to mechanical forces and biochemical cues that may originate externally, such as fluid/shear stress, osmotic and hydrostatic pressure, cyclic strain, amplitude variations, substrate stiffness, or internally through cytoskeletal contractility.^13–15^ These mechanical and biochemical inputs act in a highly coordinated manner, forming an integrated regulatory network that governs MSC fate and osteogenic commitment.

**Figure 1.**
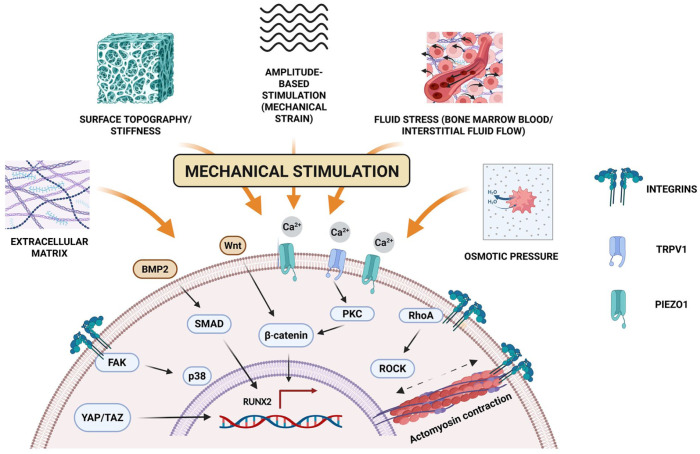
Mechanical cues and mechanotransduction pathways regulating MSC osteoblastogenesis. Schematic illustration of how distinct mechanical stimuli, such as surface topography/stiffness, extracellular matrix, fluid shear stress, osmotic pressure, and amplitude-based stimulation or mechanical strain, are sensed by mechano-sensitive structures, that is, integrins, stretch-activated ion channels, PIEZO1, and TRPV1. The stimulation triggers intracellular Ca^2+^ influx and activates FAK, PKC, and MAPK signalling pathways, including p38, thereby promoting F-actin cytoskeletal reorganisation and contraction via RhoA-ROCK signalling. Integrin-cytoskeleton coupling and Ca^2+^ influx also induce Wnt/β-catenin signalling, while mechanical cues alone enhance BMP2-SMAD activation and promote YAP/TAZ nuclear translocation in response to substrate stiffness. Collectively, these pathways converge on osteogenic transcriptional programming, which includes RUNX2 activation, to drive mesenchymal stem cell osteoblastogenesis.

Mechanical cues are sensed by mechanosensitive structures, including membrane deformation or stretch-activated ion channels (e.g. potassium channel subfamily K member 2 (KCNK2), PIEZO1/2, and transient receptor potential (TRP) channels), integrins, and actomyosin cytoskeletal components.^14,15,18,19^ Activation of these mechanosensitive structures triggers rapid calcium influx and downstream signalling processes, including activation of focal adhesion kinase (FAK), protein kinase C (PKC), ROCK, and mitogen-activated protein kinases (MAPKs) such as p38 and extracellular signal-regulated kinase 1/2 (ERK1/2).^14–23^ RhoA is a GTPase enzyme that stimulates ROCK to modulate myosin II activity,^18–20^ enhances tension in actin stress fibres,^21–23^ and regulates cell spreading and migration via lamellipodial projections (Figure 1).^19–21^

Integrins, on the other hand, are transmembrane heterodimeric glycoproteins composed of non-covalently associated α and β-subunits.^19^ They also participate in cytoskeletal reorganisation/contraction and focal adhesion stabilisation, which further propagate mechanotransduction signals (Figure 1).^24^

The primary cilium, a solitary cellular appendage extending from the cell surface, serves as a specialised mechanosensor, responding to fluid shear stress to regulate cAMP-mediated hedgehog signalling, influencing murine BM-MSC (mBM-MSC) osteogenic differentiation in vitro.^25^ Mechanical stimulation promotes localisation of G-protein-coupled receptor 161 (GPR161) to the primary cilium, activating adenylyl cyclase 6 (AC6)-mediated cAMP signalling. This, in turn, modulates the hedgehog (Hh) pathway to drive MSC osteogenic differentiation.^25^ In primary cilium-mediated mechanotransduction, calcium ions act as secondary messengers. Human cell-based studies indicated that mechanical forces increased intracellular calcium levels, which in turn induced osteoblast formation.^26^

**Figure 2.**
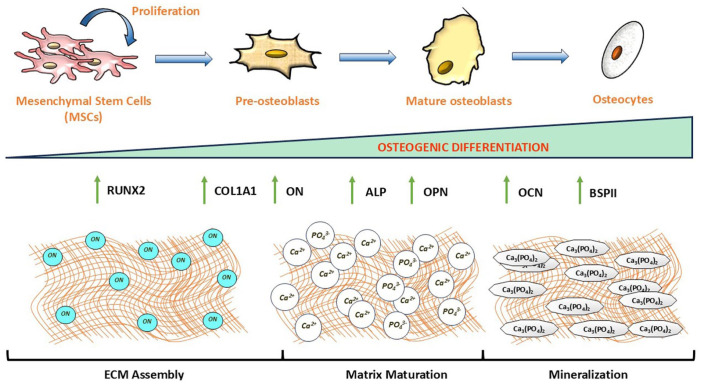
Summary of MSC osteogenic differentiation. MSCs proliferate robustly during the initial phases of osteogenic differentiation to increase their population. As they commit to osteoblasts, the proliferation rate declines, and they gradually begin to express differentiation markers, such as RUNX2, COL1A1, ON, ALP, OPN, OCN, and bone sialoprotein II (BSPII), across three stages: ON-induced ECM assembly, matrix maturation, and mineralisation. Ultimately, they develop into mature osteoblastic cells, committing to terminally differentiated osteocytes.

Mechanical stimulation of MSCs converges on key transcriptional regulators, including YAP/TAZ Hippo pathway signalling,^27^ bone morphogenetic protein 2, small body size/mothers against decapentaplegic family (BMP2/SMAD) and canonical Wnt/β-catenin signalling,^14,18,28^ which collectively induce expression of RUNX2, the master transcription factor for osteoblastogenesis (Figure 1).^11,18,23^ RUNX2 regulates the transcription of osteogenic differentiation markers, including alkaline phosphatase (ALP), collagen type 1A1 (COL1A1), osteopontin (OPN), osteocalcin (OCN), osteonectin (ON), and others, orchestrating the three stages of bone formation: ECM assembly, matrix maturation, and mineralisation (Figures 1 and 2).^11,14,18,23^ These signalling pathways demonstrate that mechanical stimulation is translated into osteogenic gene expression through multiple converging mechanotransduction networks.

Consistent with these mechanistic findings, experimental studies have further validated the role of mechanical stimuli in regulating MSC osteoblastogenesis through these signalling pathways.^29,30^ Jiang et al.^29^ demonstrated that rat BM-MSCs (rBM-MSCs) co-cultured with human umbilical vein endothelial cells (HUVECs) under fluid shear stress enhanced osteoblastogenesis via β1-integrin triggered FAK phosphorylation, which further upregulated and activated ERK1/2 and RUNX2, leading to increased ALP activity and calcium deposition in vitro.

Similarly, Zhou et al. reported that human BM-MSCs (hBM-MSCs) cultured on stiffer mineralised collagen-glycosaminoglycan (COL-GAG) scaffolds exhibited elevated YAP/TAZ expression, promoting canonical Wnt/β-catenin signalling and osteogenic differentiation. Inhibition of YAP/TAZ lowered β-catenin activation, RUNX2, COL1A1 and ALP expression, effects that were further associated with alterations in FAK phosphorylation and αV and α5 integrin expression in vitro.^30^

Furthermore, integrin-mediated interactions with non-collagenous ECM glycoproteins, such as vitronectin, osteopontin, bone sialoproteins, fibronectin, and osteolectin, govern MSC osteogenic differentiation via RGD-binding integrins such as α_v_β_3_, α_5_β_1_, α_11_β_1_, encouraging Wnt signalling and maintaining skeletal mass.^31–34^ Together, mechanical cues and integrin-ECM interactions converge to regulate transcriptional programs that drive MSC commitment, differentiation and mineralised matrix formation.

## Effect of mechanical loading on osteoblast formation

Bone homoeostasis is a tightly regulated process that maintains skeletal integrity through a coupled balance between bone formation by osteoblasts and resorption by osteoclasts. Mechanical loading is a central regulator of this physiological balance, ensuring that bone mass and architecture adapt to functional requirements.^35^

In the absence of sufficient mechanical loading, MSCs preferentially become quiescent or adopt adipogenic phenotypes, however, appropriate mechanical input biases MSC fate towards osteoblastogenesis, thereby sustaining osteogenic capacity and structural rigidity.^35,36^ Acknowledging this concept, previous reports have demonstrated that mechanical loading drives osteoblastogenesis while suppressing adipogenesis. For example, David et al. indicated that bovine bone marrow-derived MSCs (bBM-MSCs) cultured on COL1A1 and subjected to cyclic mechanical stretch and compression exhibited significant upregulation of RUNX2 and OCN, accompanied by downregulation of peroxisome proliferator-activated receptor gamma (PPARγ), a critical adipocyte-specific marker. The osteogenic lineage commitment was further confirmed by a noticeable increase in ALP activity in vitro.^35^

Another study unveiled that a very low-intensity mechanical vibration combined with cyclic hypoxia (3% oxygen) synergistically influences hBM-MSCs.^36^ Preconditioning with these stimuli activated Wnt/β-catenin and enhanced upregulation of early osteogenic differentiation markers, hence biasing MSCs more towards osteogenic commitment in vitro. It was indicated that mechanical vibration, alone or combined with hypoxia, further promoted matrix mineralisation, while simultaneously repressing adipogenic differentiation.^36^

These key findings highlight the therapeutic perspective of controlled mechanical or combined with oxygen tension cues to favour osteoblastogenesis over adipogenesis. At the mechanistic level, mechanical loading induces osteoblast formation by regulating cytoskeletal tension and mechanotransduction pathways that control nuclear translocation of transcriptional co-activators or mechanotransducers.^37–39^ Kim et al.^37^ demonstrated that cyclic mechanical stretch enhances ROCK-dependent cytoskeletal contractility, promoting nuclear YAP translocation and triggering osteoblastogenesis in mBM-MSCs in vitro. Similarly, rBM-MSCs cultured on stiff polyacrylamide (PAAm) or polydimethylsiloxane (PDMS) substrates and exposed to appropriate cyclic strain exhibited elevated ALP activity and negligible lipid formation compared to softer substrates in vitro.^38^ These effects were attributed to high intracellular tension and elevated RhoA signalling on stiff matrices, suggesting osteogenic potential over adipogenic differentiation.^38^ Further supporting the role of mechanical loading in bone homoeostasis, stiff substrates have been shown to enhance α_2_ integrin-mediated adhesion and activate focal adhesion kinase (FAK), ERK1/2 and ROCK signalling pathways, which led to the upregulation of osteogenic markers COL1A1 and OCN in vitro.^39^ Key representative experimental studies examining the effects of mechanical loading, substrate stiffness, and combined biophysical cues on MSC osteoblastogenic and adipogenic differentiation are summarised in Table 1.

**Table 1.** Mechanotransduction pathways in MSC osteoblastogenesis.

Mechanical stimulus	Model system	Downstream signalling pathways and osteogenic outcome	Reference
Fluid shear stress	mBM-MSCs	• Primary cilium (GPR161, AC6), cAMP, Hh signalling.	Johnson et al.^25^
		• Enhanced osteogenic differentiation.	
Fluid shear stress	rBM-MSCs + HUVEC co-culture	• β1-integrin, FAK, ERK1/2.	Jiang et al.^29^
		• Increased RUNX2 expression, ALP activity and calcium deposition; enhanced osteoblastogenesis.	
Stiff mineralised COL-GAG scaffold	hBM-MSCs	• YAP/TAZ, canonical Wnt/β-catenin signalling.	Zhou et al.^30^
		• Increased RUNX2, COL1A1, and ALP; osteogenesis was suppressed when YAP/TAZ was inhibited, following alterations in αV and α5 integrin expression.	
Cyclic mechanical stretch	bBM-MSCs and mBM-MSCs	• RhoA/ROCK-dependent cytoskeletal contractility, nuclear YAP translocation.	David et al.,^35^ Kim et al.,^37^ Gungordu et al.^38^
		• Increased RUNX2 and OCN, ALP activity; reduced PPARϒ expression and negligible lipid formation.	
Low-intensity mechanical vibration + cyclic hypoxia (3%)	hBM-MSCs	• Wnt/β-catenin signalling.	Camacho-Cardenosa et al.^36^
		• Increased osteogenic commitment; repressed adipogenic differentiation.	
Stiff PAAm/PDMS substrates + cyclic strain	rBM-MSCs	• RhoA signalling.	Gungordu et al.^38^
		• More ALP activity and enhanced osteogenic potential and reduced oil droplet formation.	

While these studies emphasise mechanical loading functions as a key stimulatory input in bone homoeostasis, promoting osteoblastogenesis through intracellular cytoskeletal reorganisation and mechanotransduction signalling, therefore contributing to adaptive bone formation, increasing evidence suggests that the nucleus itself functions as a mechanosensitive hub.^40^ Mechanical signals induce lamin A/C-dependent nuclear stiffening and flattening, coupled with increased enhancer-promoter interactions at osteogenic gene loci, and higher-order 3D chromatin reorganisation, including A/B compartment switching, topologically associated domain (TAD) that segregate osteogenic programs from non-osteogenic genes, thereby stabilising osteogenic fate through mechanically driven nuclear structural alterations.^40^

A feature of MSCs, termed cellular mechanical memory, facilitates the linkage between past mechanical stimuli and cytoskeletal and epigenetic changes.^41^ These memories prime MSCs to retain their osteogenic commitment even after mechanical cues cease. Mechanistically, this is supported by epigenetic imprints, including enhanced histone modifications (H3K4 trimethylation and H3K9 acetylation) at osteogenic gene loci and reduced DNA methylation at the promoter regions of osteogenic differentiation markers, thereby stabilising their expression.^41^ Simultaneously, MSCs exhibit sustained cytoskeletal reorganisation, such as persistent stress fibre formation, lasting integrin clustering, and stabilised focal adhesions, which help reinforce mechanotransduction signalling pathways and collectively sustain osteogenic differentiation.^41,42^ Furthermore, nuclear localisation of YAP persists even after transferring and exposing MSCs from a stiff to a soft substrate, exhibiting retention of mechanical memory.^41^ Thus, the mechanotransductive stimulus is remembered by cells and shapes their activity and fate.

## Physiological mechanical loading regulates bone matrix homoeostasis

The physiological red bone marrow constitutes a highly viscous and dynamically mechanical microenvironment in which resident BM-MSCs are widely exposed to physiologically relevant forces or loading, including hydrostatic pressure, osmotic pressure, tensile load, and viscosity-mediated shear stress.^43–45^ These mechanical cues not only act in isolation but also operate alongside diverse biochemical, hormonal, and inflammatory signals to shape MSC behaviour within the bone marrow niche. Importantly, mechanical loading provides spatial and microenvironmental regulation that significantly influences matrix turnover and biases BM-MSC osteogenic fate outcomes.^46^ Under suitable physiological loading, BM-MSCs activate mechanotransduction pathways channelling through integrins, cytoskeletal tension, and ion channels, culminating in the upregulation of matrix metalloproteinases (MMPs), such as MMP-2 and MMP-9.^43,47^

**Figure 3.**
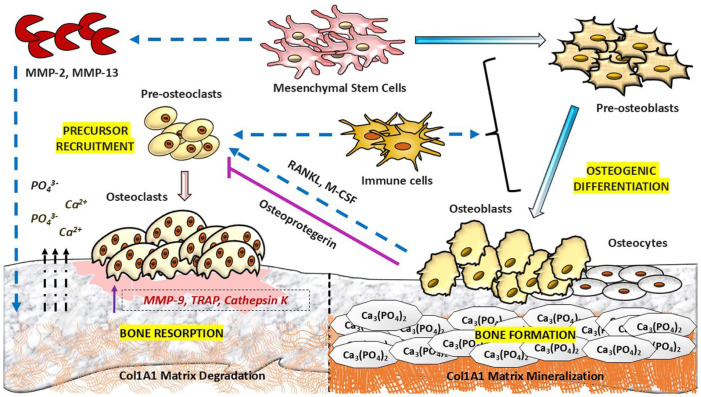
Mechanical stimulation and bone remodelling. Upon physiological mechanical loading, mesenchymal stem cells are activated and secrete matrix metalloproteinases, including MMP-2 and MMP-13, contributing to initial extracellular matrix remodelling. Concurrently, mesenchymal stem cells undergo osteoblastogenesis, committing to COL1A1-expressing pre-osteoblasts, which further mature into osteoblasts. Apoptotic or aged osteocytes and osteoblasts increase expression of RANKL and M-CSF, which interact with RANK and c-Fms receptors on osteoclast progenitors, promoting their differentiation into mononuclear pre-osteoclasts and subsequent fusion into multinucleated mature osteoclasts. Mature osteoclasts adhere to resorption sites and secrete enzymes, including MMP-9, TRAP, and cathepsin K, leading to degradation of the mineralised matrix and release of calcium and phosphate ions. During remodelling, osteoblasts can terminally differentiate into osteocytes following matrix mineralisation. The activity of both osteoblasts and osteoclasts is further modulated by mechanically responsive immune cells within the bone microenvironment.

This mechanically associated MMP activity contributes to the localised degradation of existing or aged ECM, facilitating matrix renewal and creating appropriate conditions for MSC osteogenic induction and deposition of a newly synthesised COL1A1-rich matrix.^43^ The process is tightly regulated through feedback loop mechanisms, including the expression and suppressive activity of tissue inhibitors of metalloproteinases, for example, TIMP-2, ensuring controlled, site-specific remodelling and preventing excessive matrix breakdown.^43^ Importantly, dysregulation of this MMP-TIMP balance can impair tissue homoeostasis and contribute to pathological remodelling.^43^ Mechanical cues also influence the coupling between bone formation and resorption by modulating signalling within osteoblastic cells and osteocytes. Aged, apoptotic, or mechanically stressed osteocytes, alongside mature osteoblasts, secrete soluble osteoclastogenic factors, comprising receptor activator of nuclear factor κB ligand (RANKL) and macrophage colony-stimulating factor (M-CSF), together with systemic and local biochemical regulators (Figure 3).^48–50^ These factors bind to their respective receptors, RANK and colony-stimulating factor 1 (CSF1 or c-fms), on monocyte/macrophage-derived osteoclast progenitor cells,^51,52^ promoting their survival, proliferation, and commitment to tartrate-resistant acid phosphatase (TRAP)-expressing mononuclear pre-osteoclasts under the transcriptional control of nuclear factor of activated T cells c1 (NFATc1).^53,54^

Within the RANKL-M-CSF gradient, pre-osteoclasts undergo fusion and differentiate into multinucleated mature osteoclasts (osteoclastogenesis). Mechanical signals transmitted through the bone matrix and interstitial fluid flow can further modulate osteoclast differentiation and activity, amplifying biochemical cues.^50,53^ Activated osteoclasts adhere to designated resorption sites, generate an acidic microenvironment suitable for the secretion of NFATc1-regulated enzymes, including TRAP, MMP-9, MMP-14, cathepsin K, and carbonic anhydrase 2,^55,56^ resulting in degradation and demineralisation of the aged matrix and the release of calcium and phosphate ions into the blood circulation.^57^ This coordinated enzymatic activity, including MMPs, is central to bone matrix turnover, and its dysregulation is a key contributor to pathological bone loss. Interestingly, similar matrix-remodelling mechanisms have also been observed in bone-associated anomalies such as osteosarcoma, where tumour cells utilise the mechanically dynamic bone microenvironment to enhance invasive potential. Mechanotransduction pathways activated by ECM stiffness and other collagen-rich substrates can upregulate MMP expression, particularly MMP-2 and MMP-9, thereby triggering ECM degradation and tumour cell migration.^58^ Furthermore, impaired mechanotransduction in osteosarcoma can amplify proteolytic activity and disrupt normal bone matrix remodelling, contributing to increased matrix breakdown and tumour progression.^58^

Subsequently, osteoblasts formed from mechanically primed BM-MSCs terminally differentiate into osteocytes and secrete non-collagenous proteins such as OPN and OCN, which support structural refinement of the newly deposited COL1A1 matrix and osteogenic mineralisation.^34^ Collectively, bone remodelling emerges as a coupled, multifactorial process in which mechanical loading not only acts as an exclusive modulator but also provides spatial, temporal, and contextual regulation that integrates matrix remodelling, osteogenic differentiation, and osteoclast-mediated resorption (Figure 3).

## Role of soluble and intracellular factors and immune cells in bone homoeostasis

Although pertinent to bone resorption, osteoclast activity is tightly balanced by osteogenic differentiation through regulatory networks strongly influenced by mechanical cues rather than soluble factors alone. Mechanical loading acts as a strong regulator that biases fundamental signalling axes, most notably the RANKL-osteoprotegerin (OPG) balance, therefore determining whether osteogenic or resorptive programmes predominate at a given site.^59^ OPG, an osteoblast-derived decoy receptor for RANKL, represents a central mechanosensitive checkpoint.^59^ Mechanically stimulated osteoblasts upregulate OPG, limiting RANKL-RANK interactions on osteoclast progenitors and suppressing osteoclastogenesis, ultimately favouring bone mass formation.^60,61^ Hormonal regulation overlaps with this mechanical regulation. Oestrogen, a steroid hormone, suppresses RANKL-induced osteoclastogenesis in part by upregulating OPG in osteoblasts, while also positively regulating cytoskeletal organisation and RhoA/ROCK-dependent mechanotransduction.^62^

**Figure 4.**
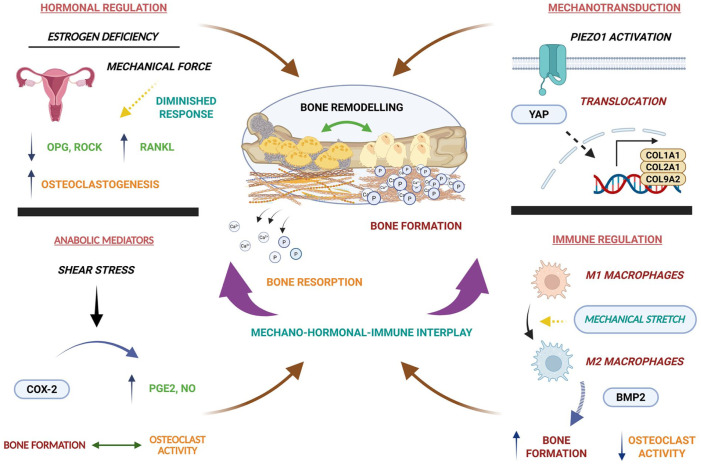
Integrated regulation of bone remodelling by mechanical, soluble, intracellular, and immune factors. Schematic representation of bone remodelling regulated by the dynamic interplay of soluble hormonal, intracellular signalling, inflammatory mediators, and immune cell-mechanical interactions. Oestrogen deficiency impairs mechanobiological response, often leading to reduced sensitivity to mechanical forces, which shifts the OPG/RANKL/RANK balance towards increased osteoclastogenesis, promoting bone resorption despite mechanical loading. Mechanical stimulation activates mechanotransduction pathways such as PIEZO1-YAP, triggering the expression of COL1A1, COL2A1, and COL9A2-like osteogenic markers. Soluble anabolic mediators, including PGE2 and NO induced by shear stress, modulate both osteogenic and osteoclastogenic responses, while immune cell polarisation influences remodelling outcomes, with macrophages polarised from a pro-inflammatory (M1) to an anti-inflammatory (M2) state secrete BMP2 via mechanical stretch-driven YAP translocation, which favours bone formation.

In oestrogen-deficient conditions, such as post-menopause, this synergy between mechanical and hormonal responses is disrupted, therefore, mechanically stimulated osteoblasts exhibit reduced OPG and ROCK expression, resulting in a heightened osteoclastogenic bias despite ongoing mechanical input, thereby predisposing bone to considerable resorption.^63^ This signifies that the protective effects of mechanical signals can depend on intact hormonal signalling (Figure 4).

Mechanical forces further regulate osteoblast-osteoclast coupling by controlling the secretion and efficiency of anabolic mediators. Fluid shear stress activates cyclooxygenase-2 (COX-2)-dependent transcriptional activity in osteoblasts, leading to production of prostaglandin E2 (PGE2) and nitric oxide (NO).^64^ Both PGE2 and NO act as load-sensitive mediators whose anabolic influence is enhanced under physiological mechanical stimulation, contributing to enhanced bone formation and indirect impediment of osteoclast activity.^64^ At the direct mechanotransduction level, the stretch-activated ion channel, PIEZO1, has emerged as a prominent node coupling physical forces to transcriptional control of bone remodelling. PIEZO1 activation promotes YAP nuclear translocation and downstream expression of collagen 2A1/9A2 (COL2A1/9A2), which inhibits osteoclast formation and supports more osteogenic matrix synthesis,^65^ therefore positioning PIEZO1 as a fundamental integrator through which mechanical deformation is converted into coordinated modulation of bone formation and resorption (Figure 4).^65^

Mechanical regulation also extends to the immune cell compartment within the marrow niche.^66–69^ Macrophages exhibit pronounced mechanosensitivity, with the degree of mechanical loading determining the inflammatory phenotype and its subsequent effects on bone cells. Considerable mechanical stretch induced a shift from a pro-inflammatory M1 phenotype towards an anti-inflammatory, pro-regenerative M2 state, accompanied by enhanced YAP nuclear translocation and increased BMP2 secretion. This mechanically driven macrophage polarisation promoted osteogenic differentiation of co-cultured mBM-MSCs in vitro, highlighting immune cells as mediators of mechanical signalling in bone (Figure 4).^66^

On the contrary, excessive or pathological mechanical forces can synergise with osteoclastogenic cues, for instance, tensile loading of RANKL-stimulated monocyte-derived macrophage activates tumour necrosis factor receptor-associated factor 6 (TRAF6) and nuclear factor kappa light chain enhancer of activated B-cells (NF-κB) signalling, elevating interleukin-6 (IL-6) and tumour necrosis factor (TNF-α) production and driving osteoclastogenesis marked by increased TRAP, MMP-9 and cathepsin K expression.^67–69^

By integrating mechanotransductive pathways with hormonal and inflammatory signals, mechanical regulation ensures a subtle equilibrium between bone resorption and formation, enabling the maintenance of skeletal integrity while allowing adaptive responses to mechanical demands.

## Mechanical unloading and its impact on bone health

**Figure 5.**
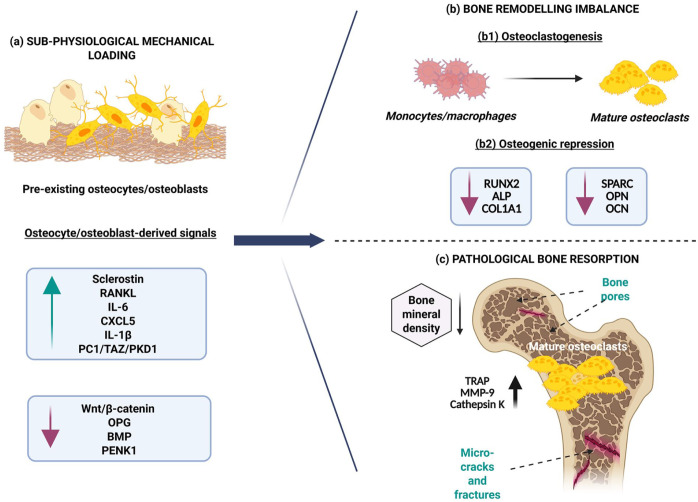
Mechanical unloading and pathological bone loss. (a) A reduction in physiological mechanical loading significantly upregulates osteoclastogenesis-specific markers in osteocytes and osteoblasts, such as IL-6, CXCL5, IL-1β, RANKL, Sclerostin, PC-1, TAZ, and PKD1. Simultaneously, it represses osteoblastogenesis-specific extracellular and intracellular factors like Wnt/β-catenin, OPG, BMP, and PENK1. (b1) These changes trigger the differentiation of monocytes/macrophages into osteoclasts, (b2) while causing an inevitable downregulation of osteogenic markers, RUNX2, ALP, COL1A1, SPARC, OPN, OCN, and more. (c) Mature osteoclasts subsequently proliferate and secrete matrix-degrading enzymes, like TRAP, MMP-9, and cathepsin K, leading to catabolic alterations in bone structure characterised by the formation of irregular pores, microcracks, and fractures, and an eventual reduction in bone mineral density. Collectively, mechanical unloading disrupts bone matrix homoeostasis by favouring excessive resorption, which often contributes to bone loss conditions such as osteopenia and osteoporosis.

Mechanical unloading represents a macroscopic loss of physiological mechanical regulation and is a well-established triggering factor for pathological bone loss, including osteopenia and osteoporosis.^70–72,84^ At the tissue or organ level, reduced mechanical stimulation disrupts the tightly regulated balance between osteogenesis and osteoclastogenesis, leading to a net increase in bone resorption (Figure 5). Clinically, this reflects a progressive decline in bone mineral density, the formation of irregular pores and microcracks within the bone matrix and an increased susceptibility to fractures.^71,72^ Osteopenia reflects an early or mild reduction in bone mass, while severe unloading promotes osteoporosis, characterised by prominent structural deterioration of the skeletal framework.^71,72^

Mechanical unloading arises from a wide range of physiological and pathological conditions that reduce skeletal loading, including prolonged bed rest, immobilisation following injury, ageing, metabolic perturbations, altered interstitial fluid flow around osteocytes, and disruption of the osteocyte-lacunar network.^71,73–75^ Altogether, these events lead to skeletal immobilisation, and the resulting bone loss associated with reduced physical activity is clinically defined as disuse osteoporosis.^71,73–75^

Parallelly, extreme unloading environments such as microgravity further illustrate the macroscopic consequences of diminished mechanical sensation or input on bone health. Astronauts subjected to prolonged microgravity experience approximately 1%–2% bone loss, particularly in weight-bearing bones such as the distal tibia, accompanied by impaired calcium absorption.^76,77^ To examine these effects, earth-based unloading models, including hindlimb or tail-suspension in rodents, have been widely employed to recapitulate the skeletal consequences of reduced loading, inactivity, immobilisation and bed rest.^78–80^

At the cellular level, mechanical unloading compromises osteoblast survival and osteocyte viability. Early osteoblast and osteocyte apoptosis within their lacunae are prominent features of unloading-induced bone loss, further intensifying the imbalance in bone remodelling.^71^ Osteocytes, as primary mechanosensors, respond to unloading by altering their signalling output, thereby influencing both osteoblast and osteoclast activity.^71^ One hallmark molecular response to unloading is the significant upregulation of sclerostin, a glycoprotein encoded by the SOST gene and predominantly produced by apoptotic osteocytes and osteoblasts. Upregulated sclerostin obstructs canonical Wnt/β-catenin and BMP signalling by interacting with low-density lipoprotein-related proteins 5 and 6 (LRP5/6), suppressing MSC osteogenic differentiation and osteoblast activity.^81^ This shift favours an increased RANKL/OPG ratio, heightened osteoclastogenesis and elevated TRAP secretion, a phenomenon widely observed in postmenopausal osteoporotic patients (Figure 5).^81–83^

Consequently, mechanical unloading reshapes the bone marrow microenvironment and MSC secretome. Wang et al.^84^ demonstrated that insufficient or sub-physiological mechanical loading disrupts bone remodelling by altering the MSC secretome towards a pro-inflammatory phenotype, thereby favouring osteoclastogenesis over osteoblastogenesis in mouse trabecular bone. Reduced mechanical stimulation via hindlimb unloading caused rBM-MSCs to secrete elevated levels of pro-inflammatory cytokines, consisting of IL-6, CXCL5, and IL-1β, alongside increased RANKL and decreased OPG concentrations within the bone marrow. These changes promoted a significant expansion of osteoclast progenitor-forming CD11b^+^Ly6C^+^ monocytes in peripheral blood circulation, both in vitro and in vivo, and were supported by the upregulation of osteoclast-associated pathway genes.^84^ More precisely, activated monocytes and macrophages are known to secrete pro-inflammatory cytokines that suppress MSC osteoblastogenesis, further encouraging catabolic remodelling during unloading (Figure 5).^85^

In clinical settings, similar mechanical unloading can occur locally around orthopaedic implants due to the stress shielding effect, particularly with rigid and stiff materials such as titanium alloys, including Ti-6Al-4V.^86^ The high stiffness of these implants relative to bone reduces the physiological load transmitted to surrounding bone, therefore leading to localised bone resorption and altered MSC behaviour that resembles the pro-inflammatory secretome changes observed under unloading conditions, suggesting that bone implant materials can directly modulate MSC-mediated bone matrix remodelling.^86,87^ This is in accordance with Wolff’s law, which states that bone adapts to the mechanical load placed upon it, however, when rigid implant materials take over the majority of the mechanical load, while the surrounding bone experiences reduced loading, this augments the likelihood of osteoclastogenesis-directed bone resorption, which has been considered a leading cause of hip replacement failures.^88^ Orthopaedic implants are typically much stiffer, hence, the surrounding bone receives insufficient mechanical stimulation.^88^

At the molecular signalling level, unloading impairs multiple mechanosensitive pathways that normally coordinate bone homoeostasis. Puri et al.^89^ reported that reduced mechanical loading during bed rest led to downregulation of pro-enkephalin 1 (PENK1) in mouse osteoblasts, which was accompanied by a large increase in sclerostin expression and reduced secretion of COL1A1 and Wnt3a. Osteoblasts under these conditions exhibited impaired metabolism, diminished ALP activity and reduced mineralisation capability in vitro. Although PENK1 was previously identified as a neuropeptide involved in bone remodelling and matrix mineralisation, its unloading-induced downregulation during sustained immobilisation suggests a contributory role in the progression of disuse osteoporosis (Figure 5).^89,90^

Microgravity and other unloading models have further highlighted the complexity of mechanotransduction pathways involved in bone loss. Huang et al.^91^ showed that tail suspension induced unloading upregulated polycystin-1 (PC-1), a mechanosensitive transmembrane protein encoded by polycystic kidney disease 1 (PKD1), which interacted with the mechanotransducer TAZ to enhance osteoclastogenesis in mouse bone marrow macrophages. This interaction increased TRAP-positive osteoclast formation and trabecular bone resorption in vitro and ex vivo and was associated with elevated expression of osteoclast-specific genes, including NFATc1, cathepsin K, and RANK. These molecular changes were mechanistically linked to disuse osteoporosis, rheumatoid arthritis, and Paget’s disease-like bone anomalies.^91,92^

Representative studies on mechanical unloading and its impact on bone homoeostasis are summarised in Table 2.

**Table 2.** Effects of mechanical unloading on MSC function and bone remodelling.

Unloading condition	Model system	Key signalling pathways and associated outcomes	Reference
Hindlimb unloading/reduced mechanical loading	mBM-MSCs	• IL-6, IL-1β, CXCL5, RANKL.	Wang et al.^84^
		• Decreased OPG and more osteoclastogenesis in an MSC pro-inflammatory secretome.	
Bed rest/reduced mechanical loading	Mouse osteoblasts	• Downregulated PENK1, Wnt3a and COL1A1 and diminished ALP activity.	Puri et al.,^89^ Seitz et al.^90^
		• Increased sclerostin, followed by impaired mineralisation and osteoblast function, leading to disuse osteoporosis.	
Tail suspension (microgravity model)	Mouse bone marrow macrophages and mBM-MSCs	• PC-1/TAZ signalling.	Xiao et al.,^91^ Gargalionis et al.^92^
		• Increased osteoclastogenesis via TRAP and NFATc1 expression.	
Tail suspension (microgravity model)	Transgenic mice	• Reduced CX43 signalling.	Garg et al.^93^
		• Increased RANKL/OPG ratio, porosity and trabecular bone loss.	

Coincidentally, the PC-1/TAZ/PKD1 signalling axis also promotes MSC osteogenic differentiation under mechanical loading through activation of the Janus kinase 2/signal transducer and activator of transcription 3 (JAK2/STAT3) and Akt/β-catenin pathways, reflecting on its context-dependent dual role in bone biology.^91,92^ However, the exact mechanisms by which this axis preferentially induces bone resorption during unloading remain poorly understood (Figure 5).^92^

Intracellular communication within bone is also profoundly affected by mechanical unloading. Connexin 43 (CX43), a gap junction and hemichannel protein essential for osteocyte-osteocyte or osteocyte-osteoblast communication, participates in skeletal adaptation to unloading.^93^ Transgenic mice with impaired CX43 subjected to tail-suspension-induced microgravity demonstrated considerable distal femoral trabecular bone loss, elevated RANKL/OPG ratios, and increased sclerostin expression, both in vitro and in vivo.^93^

CX43 gap junctions are required for the propagation of PGE2 signalling, a key driver of bone formation. Studies show that CX43 deletion leads to osteopenic phenotypes characterised by reduced cortical bone mineral density and increased bone porosity in vivo.^93^

Conjointly, mechanical unloading exerts its deleterious effects on bone health through a systemic cascade bridging macroscopic loss of skeletal loading, cellular dysfunction of osteoblasts, osteocytes, and immune cells, and molecular disruption of mechanosensitive signalling pathways. These coordinated events ultimately redirect bone remodelling towards excessive osteoclastogenesis and pathological bone loss, culminating in osteopenia and osteoporosis (Figure 5).

## Nanovibrational mechanical stimulation

**Figure 6.**
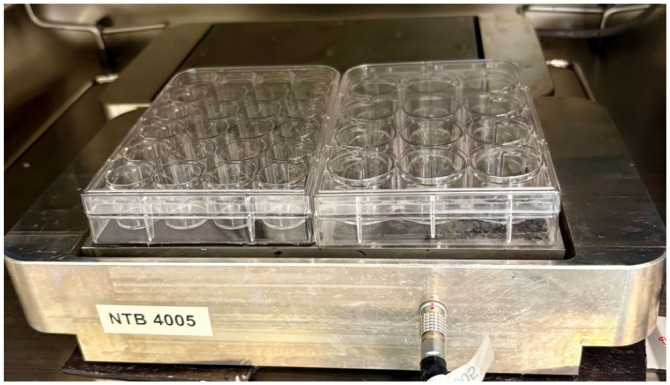
Magnetically coupled culture plastics mounted on the vibrating platform of the nanovibrational bioreactor. Cell culture plates are magnetically coupled to a nanovibrational bioreactor, which is connected to a signal generator and produces a signal output with a specific amplitude (typically 30 nm displacement) and a frequency (1000 Hz). Original photograph taken by the authors.

Given the detrimental outcomes of mechanical unloading, often resulting in bone-related pathologies,^70,71,84^ and additionally, the potential limitations of conventional chemical induction therapeutic approaches, such as poor cell differentiation specificity, cellular stress, and side effects like inflammation,^94^ there is growing interest in producing mature osteoblasts for therapeutic transplantation by directing MSC osteogenic differentiation through nanomechanical loading.^14,18,95^ The concept of nanomechanical loading, or nanovibrational mechanical stimulation, was developed as a precise, well-controlled, and scalable mechanical force that associates cellular adhesion to surfaces with intracellular cytoskeletal contraction, aiding in driving osteoblastogenesis in MSCs, which is typically based on how cells respond to their mechanical environment.^14,95^ This led to the development of a nanovibrational bioreactor, as shown in Figure 6, that operates on the principle of the reverse piezoelectric effect, generating mechanical force at nanoscale amplitudes or displacement (nm) in response to an applied electric field.^18,23,69,96^

Multiple in vitro studies have shown that defined nanovibrational regimes can reliably induce MSC osteoblastogenesis and minimise the likelihood of osteoclastogenesis.^69^ Orapiriyakul et al. suggested that nanoscale displacements of 30–90 nm applied at a frequency of 1 kHz consistently upregulated prominent early and late osteogenic differentiation markers, including RUNX2, osterix (OSX), ALP, OPN, and OCN, and certain mechanotransducers such as PIEZO2, SMAD1, and ERK1/2 in hBM-MSCs cultured on 2D substrates, accompanied by a reduced expression or marked downregulation of pro-inflammatory markers, NF-Κb, IL-1β, and TNF-α, but a moderate increase in IL-6 expression was reported.^23,95^ Similar osteogenic responses have been observed in MSCs embedded within 3D collagen gels that better reflect the native bone marrow environment, indicating that the effect is not limited to simplified culture conditions.^23^ Frequency screening investigations further demonstrate that osteogenic induction is highly specific, with 1 kHz producing the most significant responses compared to other frequencies between 1 and 5 kHz.^96^ In general, MSC osteogenic differentiation requires a controlled inflammatory environment, with pro-inflammatory marker expression confined to a regenerative scale.^97^ Certain cytokines, such as IL-6, are pleiotropic in nature and therefore can also contribute positively to bone homoeostasis through pathways such as STAT3 signalling, whereas others, including IL-8 and TNF-α, are strongly pro-inflammatory and can reduce MSC osteogenic potential.^98–100^ These cytokines may also recruit monocytes/macrophages to differentiate into osteoclasts, ultimately promoting bone resorption.^99,100^ These findings suggest that nanovibrational stimulation may shift MSCs towards a more pro-regenerative phenotype that is permissive for osteogenic differentiation.

In agreement with this, Kennedy et al. showed that hBM-MSCs co-cultured with CD14^+^ monocytes and subjected to a nanovibrational force of 40 nm at 1 kHz exhibited a marked reduction in the number of TRAP+ osteoclasts, along with optimal expression of pro-inflammatory cytokines, including IL-6 and TNF-α. Concurrently, enhanced mineralisation and the upregulation of osteoblast-specific markers, including OPN, ALP, and PIEZO1, were observed in vitro.^69^

These reports imply that the osteogenic phenotype is optimally induced at specific amplitudes and frequencies of nanovibrational force, which aligns directly with cellular responses to physiological mechanical loading, and this strongly suggests that nanovibrational loading can promote osteoblastogenesis while limiting osteoclastogenesis under controlled culture conditions in vitro. Therefore, by activating mechanosensitive pathways and ameliorating the drawbacks associated with conventional chemical induction approaches, this strategy may represent a promising platform for producing osteogenic cells and highlighting the central role of mechanical loading in maintaining bone health and promoting osteogenesis.

## Conclusion

Mechanical cues play an essential role in the modulation of MSC osteogenic differentiation, matrix remodelling, and bone homoeostasis. In this review, we have highlighted how diverse forms of mechanical loading, such as material stiffness, mechanical strain, and tensile force, activate fundamental mechanotransduction pathways such as RhoA-ROCK, BMP2/SMAD, YAP/TAZ, Wnt/β-catenin to promote osteoblast maturation, matrix secretion, and mineral balance. These intricate mechanisms concerning mechanical stimulation not only influence MSC osteoblastogenesis but also govern a delicate homoeostasis between osteoblast and osteoclast formation/activity, in dynamic coordination with various soluble secretory factors, immune cell interaction, matrix degradation and synthesis, and mineralisation. On the contrary, mechanical unloading contributes to osteoporosis-like skeletal pathological conditions, represented by extensive monocyte/macrophage osteoclastogenesis, matrix degradation, and compromised skeletal integrity. This illustrates the prominence of mechanical loading in maintaining bone matrix homoeostasis. Although considerable progress has been made in elucidating the mechanical regulation of bone matrix remodelling, several aspects remain to be comprehensively studied, for example, the robust capacity of nanovibrational bioreactor to yield mature osteoblasts requires further validation through systemic testing across a broader range of amplitudes and frequencies, and additionally, an interplay between mechanical stress, cellular respiration, and inflammation on customised material platforms, in the context of bone tissue regeneration. It should be noted that most evidence discussed in this review is derived from in vitro and preclinical studies, with limited clinical validation, which may affect direct translational applicability. By exploiting these insights, future research should aim to translate them into identifying additional biomarkers and devising affordable therapeutic approaches targeted at specific skeletal anomalies associated with mechanical unloading across a wide range of age groups, conclusively enhancing patient outcomes.
